# Psychological Treatment of Medical Patients Struggling with Harmful Substance Use

**DOI:** 10.5195/jmla.2020.991

**Published:** 2020-10-01

**Authors:** Donna Belcinski

**Affiliations:** 1 donna.belcinski@greenwichhospital.org, Sackler Medical Library, Greenwich Hospital, Greenwich, CT

Substance abuse is a problem with a long history of affecting individuals and society. In recent times, the opioid epidemic has been particularly troublesome, with many states near emergency status in their efforts to combat it. How do mental health providers working in the medical field handle opioid abusers and others with substance abuse issues?

Julie A. Schumacher and Daniel C. Williams have written a guide to help mental health providers screen for and treat substance use disorders. As part of the Clinical Health Psychology Series, *Psychological Treatment of Medical Patients Struggling with Harmful Substance Use* is a comprehensive yet concise book that explains the neurobiology of addiction and offers clear guidance on how to spot and treat it. The authors know their subject matter through personal experience, so their advice is that of experts who have treated substance abusers. They write that they have learned by asking the right questions about how to identify at-risk individuals in the clinical setting, those who are already using, and those who are steeped in a life of addiction.

The authors divided the book into two sections, one giving an overview of substance use, the other covering psychological assessment and intervention for substance use and comorbid conditions. Chapters in section one include a guide to understanding harmful substance use, sociocultural aspects of substance abuse, levels of care and standard treatments, and assessment and interventions. Section two is made up of chapters addressing assessment and treatment of the common comorbidities of depression, anxiety, and sleep dysregulation. These are challenging conditions in patients who also suffer from substance abuse. The section concludes with chapters on family treatment, relapse, and future directions for treating substance abusers. The chapters end with clinical vignettes that help to show how to put the recommendations into practice.

While a mere 162 pages long, the book is well researched and written as a quick reference guide for the struggling practitioner who needs help assessing those patients with substance use disorders. The 26 pages of references can certainly be used to direct anyone wishing for more information on this timely subject. I recommend *Psychological Treatment of Medical Patients Struggling with Harmful Substance Use* for both practitioners, and medical librarians wishing to round out their collections in this practice area.

